# The crop wild relative *Fragaria vesca* as source of resistance against strawberry anthracnose

**DOI:** 10.1111/plb.70087

**Published:** 2025-08-07

**Authors:** C. Rose, I. Vogt, A. Reineke, J. Ludwig‐Müller, P. Nick, C.‐M. Geilfus

**Affiliations:** ^1^ Department of Soil Science and Plant Nutrition Geisenheim University Geisenheim Germany; ^2^ Faculty of Agriculture/Environment/Chemistry HTW Dresden – University of Applied Sciences Dresden Dresden Germany; ^3^ Department of Crop Protection Hochschule Geisenheim University Geisenheim Germany; ^4^ Faculty of Biology Technische Universität Dresden Dresden Germany; ^5^ Joseph Gottlieb Kölreuter Institute for Plant Sciences, Molecular Cell Biology Karlsruhe Institute for Technology Karlsruhe Germany

**Keywords:** *C. acutatum*, *Colletotrichum nymphaeae*, host–pathogen interaction, pathogen associated molecular pattern triggered immunity (PTI)

## Abstract

- The genetic diversity of *Fragaria* species *in situ* has great potential for breeding resistance in cultivated strawberries against anthracnose disease. In this study, we investigated the host–pathogen interactions of 72 *F. vesca* genotypes of various German origins to identify new resistance to *Colletotrichum*, one of the most economically important genera of pathogens in *F.* × *ananassa* cultivation.
- The host–pathogen interactions were monitored by symptomatic scoring after spray‐inoculation of *F. vesca* genotypes with monoconidial *Colletotrichum* isolates of different species recently determined in German strawberry fields.
- We observed significant variation in host–pathogen interaction, ranging from host genotypes exhibiting resistance to a single pathogen strain, to those demonstrating broad‐spectrum resistance. The most promising *F. vesca* genotype, NO 04 002, showed resistance to at least six *C. nymphaeae* strains and tolerance to other pathogen species. However, the monogenetic resistance gene *RCA2*, known from *F.* × *ananassa*, was not detectable in any of the ancestral *F. vesca* genotypes, suggesting a more basal resistance.
- Our study demonstrates the potential of a crop wild relative (CWR), namely *F. vesca*, as genetic resource for resistance to economically relevant strawberry anthracnose. Since this resistance is based on nonspecific defence mechanisms in the host, it contributes to the development of breeding strategies that are less susceptible to resistance erosion, supporting more sustainable strawberry production.

## Graphical abstract

The crop wild relative *Fragaria vesca* is a valuable resource to mine for novel resistance factors against *Colletotrichum*.

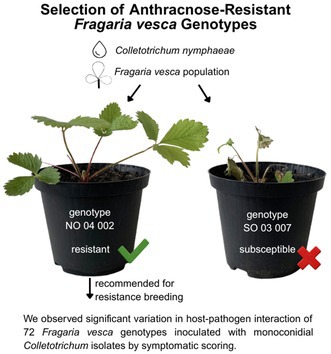

## INTRODUCTION

Strawberries (*Fragaria × ananassa*, Rosaceae) are very popular, with annual production of 8.6 million tons worldwide. However, strawberry monoculture is increasingly threatened by economically relevant diseases. The fungal disease strawberry anthracnose was first discovered in Florida, USA, caused by the pathogen *Colletotrichum fragariae* (Brooks 1931). At least 22 species complexes of *C. acutatum*, *C. boninense*, *C. coccodes*, *C. dematium*, *C. gloeosporioides* and *C. truncatum* are known as the causative pathogens (Damm *et al*. 2012; Wang *et al*. 2019; Farr & Rossman 2024). This disease has spread globally through latently infected plantlets and emerged across Europe in the 1990s (Denoyes & Baudry 1995; Laun & Fried 1996; Bobev *et al*. 2002; Nilsson *et al*. 2005; Sundelin *et al*. 2005; Garrido *et al*. 2008; Debode *et al*. 2009). Almost all parts of the plant can be affected by necrotic, brown, sunken spots, and formation of an orange conidial mucus (Howard *et al*. 1992; Laun & Fried 1996; Rose & Damm 2024). In warm and humid seasons, yield losses up to 50% or even complete plant death are common (Delp & Milholland 1980; Laun & Fried 1996; Rose & Damm 2024). To control *Colletotrichum* in strawberry, conventional methods typically involve the use of fungicides that contain azoxystrobin (BVL 2024). However, *Colletotrichum* isolates with a high level of resistance to azoxystrobin, associated with *G143A* in the cytochrome b gene, have been discovered (Luo *et al*. 2021). Fungicidal treatment is expected to soon become ineffective. Alternative crop protection measures, such as mulching, are not helpful when the plants already carry a latent infection. Currently, the only effective approach to prevent further spread of this disease is the use of certified disease‐free plant material (Gauthier & Wright 2021).

In other fruit crops, such as apple (Švara *et al*. 2024) or grapevine (Eibach *et al*. 2007), breeding for pathogen resistance can substantially contribute to reduce frequency of fungicide application, shifting the focus to plant immunity. Plants possess innate immunity, which has two levels (Nimchuk *et al*. 2003). A basal Pattern Triggered Immunity (PTI) is induced when generic motifs on the surface of the pathogen, so‐called Pathogen‐Associated Molecular Pattern (PAMP), bind as ligands to receptors on the plasma membrane of the host cell. Binding of these ligands deploys a signal cascade culminating in the accumulation of defence compounds, termed phytoalexins, but also other defence responses, such as the formation of callose plugs, oxidative burst, or the synthesis of proteins that can interfere with the pathogen (Jones & Dangl 2006). This PTI is particularly effective against necrotrophic fungi and bacteria. Isolates of *C. acutatum* and *C. fragariae* can initiate their necrotrophic (subcuticular, intramural) infection cycle in garden strawberry via a short hemibiotrophic (intracellular) phase (Curry *et al*. 2002; Wharton & Diéguez‐Uribeondo 2004). These switch off PTI by effectors and invade the host (Jones & Dangl 2006). Upon prolonged co‐evolution, the host develops a new generation of intracellular receptors, often belonging to the Nucleotide‐Binding Leucine‐Rich Receptor family (NB‐LRR), that can bind these effectors and activate a second round of Effector Triggered Immunity (ETI). This is very efficient and often involves a Hypersensitive Reaction (HR) of the attacked cell that, through its sacrifice, contains the pathogen and protects the neighbouring cells from infection (Jones & Dangl 2006).

The R proteins, encoded by NB‐LRR genes, are important triggers for diversity of defence mechanisms in cultivated strawberry (Zhang *et al*. 2016). The majority of NB‐LRRs are found in the dominant subgenome of *F. × ananassa*, derived from the diploid *F. vesca*, an ancestor of today's garden strawberry (Li *et al*. 2013; Edger *et al*. 2019). In fact, artificial inoculation of European genotypes of *F. vesca* with *C. acutatum* led to the identification of two genotypes that are resistant (Denoyes‐Rothan *et al*. 2005). As often in allopolyploids of garden strawberry, one subgenome dominates in terms of expression, while the other subgenomes progressively loose functional genes (Adams *et al*. 2003; Li *et al*. 2013). The strawberry variety ‘Capitola’ was found to be resistant to a specific *C. acutatum* strain depending on a single dominant locus, *RCA2*, controlling high level resistance (Denoyes‐Rothan *et al*. 2004, 2005).

However, it is not clear whether the fungal strains used in research for germplasm improvement were indeed *C. acutatum*, as the pathogen was determined exclusively on the basis of morphology and traditional taxonomy (Simpson *et al*. 1994; Denoyes & Baudry 1995; Denoyes‐Rothan & Guerin 1996; Denoyes‐Rothan *et al*. 1999, 2005; Lerceteau‐Köhler *et al*. 2005). Using molecular information from multiple nuclear loci revealed that, for garden strawberries in Germany, it is *C. nymphaeae*, in the *C. acutatum* species complex, that is the most important pathogen, while *C. godetiae* and *C. fioriniae* (*C. acutatum* species complex), as well as *C. lineola* (*C. dematium* species complex) are found only occasionally and episodically (Rose & Damm 2024). Interestingly, *C. acutatum* (*sensu stricto*) was not detected in this survey.

As CWR often have retained a plethora of resilience factors, they are considered as important resources for breeding (Lewers *et al*. 2007; Eikemo *et al*. 2010; Diamanti *et al*. 2014; Jiao *et al*. 2016; Qaderi *et al*. 2023; Ruthes & Dahlin 2023; Kanbar *et al*. 2024). To ensure these potentials are available for scientific research and practical breeding, the German Ministry of Agriculture initiated a national gene bank for CWRs native to Germany (Borgmann *et al*. 2020). This initiative led to storage of a seed collection comprising over 4500 accessions from more than 270 species, sourced from over 350 habitat types across the country. However, the potential of this germplasm has remained mostly untapped so far. Recently, woodland strawberries from this collection have been found to have superior cold tolerance linked to more rapid induction of a transcriptional master switch for cold hardening, and metabolic changes, such as elevated levels of tyrosol, γ‐butyric acid or proline (Kanbar *et al*. 2024).

Overall, *F. vesca* might harbour different types of resistance against *Colletotrichum* species that do not necessarily need to be ETI. Our study investigates the untapped resistance potential of 72 *F. vesca* genotypes against economical important *Colletotrichum* species through inoculation with single‐spore isolates. We explored diverse defence responses and identified promising resistance traits that may complement or even surpass existing strategies. Our findings highlight the potential of *F. vesca* as a resource for developing more resilient strawberries while supporting sustainable production practices.

## MATERIAL AND METHODS

### Plant material

A total of 3404 achenes of native *F. vesca* were selected, most of which were provided by the Genbank für Wildpflanzen für Ernährung und Landwirtschaft (Genbank WEL), Botanical Garden Osnabrück, Germany (https://www.bogos.uni‐osnabrueck.de/Genbanken/WEL‐Genbank.html). These accessions mainly originate from southwest (SW), southeast (SO), northwest (NW) and parts of northeast (NO 01) Germany, supplemented by our own collections (NO 02, 03, 04) (Table S1). The breeder code is made up of federal territory, local area number, and genotype number.

Seeds with visible vitality were sown in seed trays on a standardized nutrient‐poor propagation substrate (Stender, Schermbeck, Germany) mixed with 20% sand and germinated in an indoor foil greenhouse (95% humidity, 18–24°C, 16 h/ 8 h photoperiod at 2000 lux, with gradual acclimatization after the first leaflets developed) in winter 2019. After 3 weeks, the strawberry plantlets were pricked out into pricking trays containing the same propagation substrate as above. Parasitic nematodes *Steinernema feltiae* (Katz Biotech, Baruth, Germany) were poured over the young seedlings to control larvae of fungus gnats (*Sciaridae*). Next, the plants were potted into cultivation pots (diameter 11 cm) in propagation substrate as above as soon as three leaves had developed. The plants were cultivated in a greenhouse (80% humidity, 16–18°C, 16 h/8 h photoperiod at 2000 lux, watered weekly with rainwater). In spring 2020, young plants were transferred outdoors and potted in culture substrate consisting of 2:1 Compo Bio Kräuter‐ und Anzuchterde (Compo, Münster, Germany) and 30% sand (Mobau Müller, Bannewitz, Germany). Plants were re‐potted as mother plants once a year with the addition of 5 g Compo Bio NaturDünger Guano (Compo). A visually healthy test population of 72 *F. vesca* genotypes, with three genotypes per locality, was selected over 1 year to represent genetic diversity for the inoculation experiments with *Colletotrichum*. Plants were propagated via stolons from mother plants. They were cultivated on the same culture substrate as above, without fertilization, and watered with rainwater supplemented with tap water during hot periods. Two‐month‐old plants were acclimatized in the experimental environment for 2 weeks before the tests started.

### Inoculum and inoculation

The culture collection of Senckenberg Museum of Natural History Görlitz, Görlitz, Germany (GLMC) provided the *Colletotrichum* strains *C. nymphaeae* (GLMC 2445, GLMC 2449, GLMC 2455, GLMC 2552, GLMC 2595, GLMC 2600), *C. godetiae* (GLMC 2589, GLMC 2590), *C. lineola* (GLMC 2587) and *C. anthrisci* (GLMC 2616), which served as inocula (Rose & Damm 2024). The strains were stored as single spore isolates and propagated on oatmeal agar medium (OA; Crous *et al*. 2019) at 20° C under near UV light for 10 days. Conidia were harvested by adding 10 mL sterile distilled water to each OA culture, and swirling thoroughly. The conidium suspensions were adjusted to 2.10^6^ conidia mL^−1^ and applied at an average of 50 mL per plant. Aboveground plant parts were spray inoculated until run off using a hand pressure sprayer (Emil Lux, Wermelskirchen, Germany). Control plants were included in each experiment and sprayed with the same amount of sterile distilled water.

### Preliminary selection experiments

**Fig. 1:**
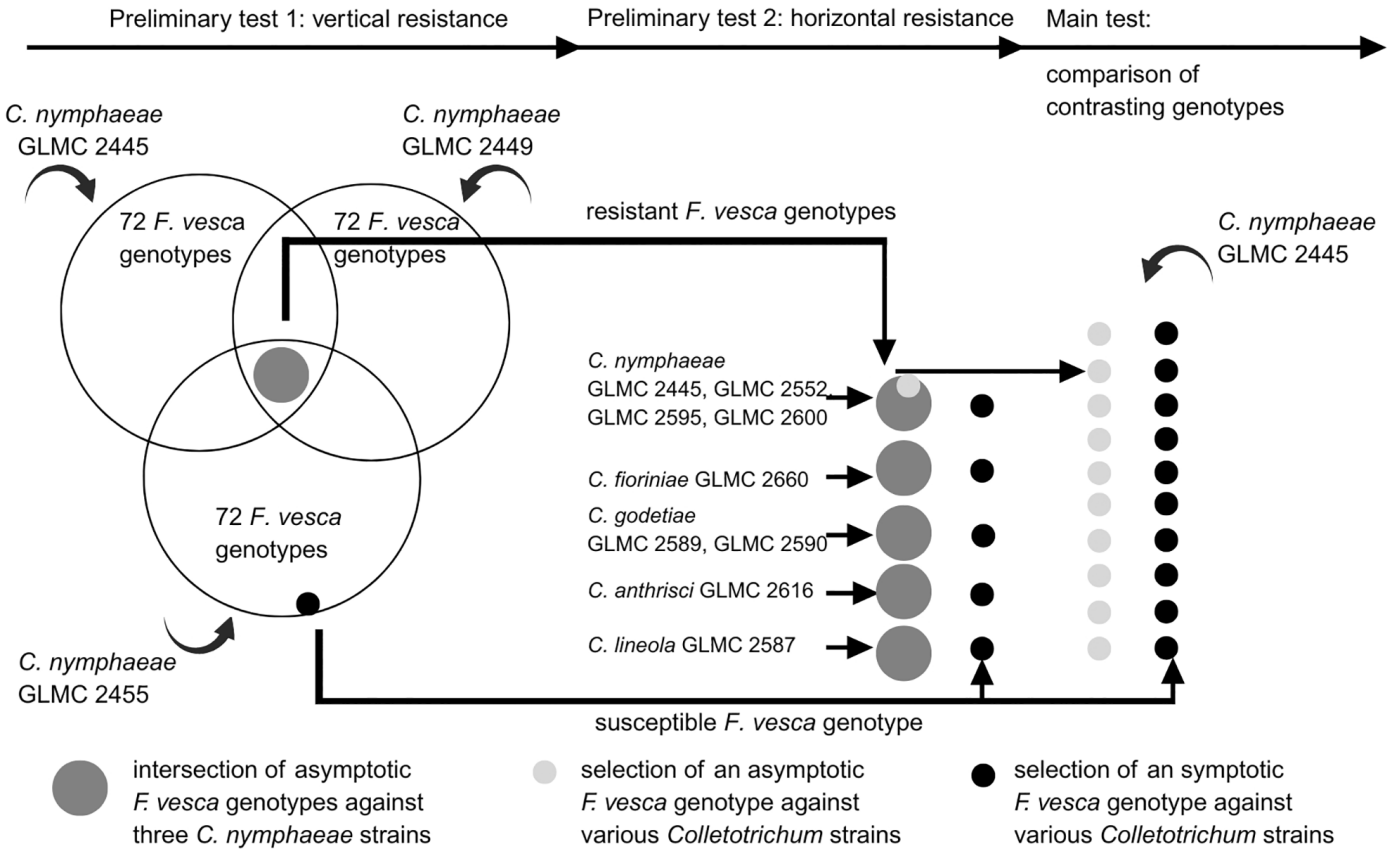
Set up of the inoculation experiments on host–pathogen interactions between *Fragaria vesca* genotypes and different *Colletotrichum* strains with previous selection steps and main experiment for comparison of contrasting genotypes.

To assess vertical resistance, we inoculated the *F. vesca* population of 72 genotypes with three closely related *C. nymphaeae* strains (GLMC 2445, GLMC 2449, GLMC 2455) as described above (Fig. 1). Since only a limited number of equivalent offshoots can be produced from a single mother plant, each strain of inoculum was tested in one replica. The plants were incubated for 21 days (95% humidity, 22–25°C day/18°C night, 16 h/ 8 h photoperiod) in separate foil greenhouses. To assess horizontal resistance, seven healthy *F. vesca* genotypes and one susceptible genotype, all identified from preliminary vertical resistance screening, were spray‐inoculated and incubated as above, with one replica each (i.e. *n* = 2), in parallel test units with *C. nymphaeae* (GLMC 2445, GLMC 2552, GLMC 2595, GLMC 2600), *C. godetiae* (GLMC 2589, GLMC 2590), *C. anthrisci* (GLMC 2616) or *C. lineola* (GLMC 2587) (Fig. 1). Plants were evaluated visually by symptom scoring and divided in categories: (1) no symptoms of infection, (2) single focus of infection, (3) strong infection on different parts of the plant, and (4) died because of infection. Genotypes without symptoms (category 1) were regarded as resistant, those with mild symptoms (category 2) as tolerant, those with severe symptoms (category 3) as susceptible, and those dying (category 4) as highly susceptible. Conidia formation on single leaflets was stimulated in a humidity chamber under incubation conditions as above for re‐isolations and identification. A camera (iPad A1823; Apple Retail Germany, Munich) was used for photo documentation. Images were brightened using the image editing programs Photoshop (Adobe I Delaware, USA), Canva (Kippax, AUS) (https://www.canva.com) and Photopea (Czech Republic) (https://www.photopea.com).

### Comparative selection experiments

Two contrasting *F. vesca* genotypes, NO 04 002 (resistant) and SO 03 007 (susceptible), identified from the above preliminary experiments, were prepared, inoculated, and incubated as above with *C. nymphaeae* GLMC 2445 (Fig. 1). To statistically evaluate the test data, we used 10 replicas each (i.e. *n* = 10).

#### Inoculation test

Plants were incubated in foil greenhouses for at least 5 days, followed by 10 days outdoors (August 2022), before being evaluated for symptoms and categorized as described above. The ordinal data were analysed using the non‐parametric Mann–Whitney *U*‐test for two independent samples at a significance of *α* = 5%.

#### Resistance staining with nitro blue tetrazolium (NBT)

To evaluate superoxide production, the NBT test was carried out as part of the comparative inoculation test. One leaflet per plant was harvested after 12 h of incubation, placed on a small glass and doused completely with 40 mL yellow staining solution (50 mM potassium phosphate buffer (pH 7.8), 0.1% NBT, 10 mM sodium azide). Open glasses were vacuum infiltrated in a desiccator (2 min, 0.8 bar; Vario PC 2001, Vacuubrand, Wertheim, Germany) to facilitate absorption. Covered glasses were kept for 2 h in darkness. A purple colour change of the staining solution is expected when NBT is reduced to formazan by superoxide radicals in oxidatively stressed plants. Formazan‐positive cells should turn blue. The results become visible when samples are decolorized by flushing out chlorophyll with 96% ethanol after NBT treatment. The ethanol bath was renewed daily for at least 3 days until complete loss of colour. Transmitted light (LD LED Searchlight 10 W, 1000 lm; Lighting EVER, Eschborn, Germany) was used for photo documentation. Lesion sizes were determined by counting blue coloured unit squares on a grid placed over the scaled photos. The metric data of superoxide‐producing areas were compared pairwise, one‐sided, using a *t‐*test of two independent samples and *α* 5% (Excel Microsoft 365). In cases of large differences in the variances of the data, we later used a *t‐*test with unequal variances.

#### Salicylic acid (SA) extraction and measurement of hormone levels

To determine the plant‐initiated immunity, a biochemical analysis of SA content was carried out as part of the comparative inoculation test. After 12, 36 and 60 h post‐inoculation (hpi), a single leaflet of each plant was harvested, from which one leaf disk of 1 cm diameter was cut out. A mixed sample of 10 leaf discs per genotype was frozen in liquid nitrogen and ground to a fine powder. To extract glycosidically bound SA, 0.7 mL 90% methanol (internal standard: 500 pmol ortho‐anisic acid) and 1.4 mL 100% methanol were added. This extract was transferred to a 2 mL Eppendorf tube and centrifuged for 10 min at 14000 rpm. The clear solution was taken out and the solvent removed on a rotary evaporator. The aqueous, oily residue was taken up in 1 mL 5% trichloroacetic acid and briefly homogenized in an ultrasonic bath. The sample was then divided into two samples of 500 μL (sample A and B). To determine total SA, samples of A were mixed with 70 μL concentrated HCL before extraction and placed in a water bath at 96°C for 1 h. To extract free SA, samples were cooled in an ice bath and stirred twice with 1 mL ethyl acetate/cyclohexane/isopropanol (EE/Chx/IPA; 50:50:0.5) in a vortex laboratory stirrer. To achieve better separation of phases, the mixtures were briefly centrifuged. The combined organic phases were concentrated on a rotary evaporator, and the residue was taken up in 200 μL methanol. The samples were stored at −20°C. The endogenous SA in leaf material was determined by gas chromatography–mass spectrometry (GC–MS) following Campanella *et al*. (2003). The samples were derivatized by methylation using trimethylsilyldiazomethane in diethyl ether according to Migowska *et al*. (2010). The extracts were separated on a Phenomenex ZB‐5 column (30 m × 0.25 mm × 0.25 μm) (Phenomenex, Darmstadt, Germany) using He carrier gas at 1 mL min^−1^. Peaks were measured according to retention time of SA and their areas integrated based on the exogenous standard o‐anisic acid.

#### Qualitative polymerase chain reaction (PCR) for resistance gene *RCA2*

We tested *F. vesca* genotypes (NO 02 001, NO 04 002, SO 01 022, SO 02 001, SO 03 007, SO 04 008, SO 05 012, SO 06 020, SW 06 007) for presence of the resistance gene *RCA2* known from *F. × ananassa* ‘Capitola’ (Lerceteau‐Köhler *et al*. 2005). Plant material of ‘Capitola’ was provided by GRIN‐Global (Davis, CA, USA) and used as positive control. Furthermore, the economically important strawberry varieties ‘Elsanta’ and ‘Asia’ (Obstbau R. Rüdiger, Dresden, Germany), were integrated into the experiment. DNA was isolated using dried leaf material and CTAB (Murray & Thompson 1980). PCR reactions were performed in 20 μL final volume containing: 20 ng genomic DNA, AccuStart II PCR SuperMix (Quantabio, Beverly, MA, USA) (Table S2), 0.2 μM of each primer (STS‐*RCA2*_240‐F/‐R, EMFv020‐F/‐R) and a PCR ‐ompatible loading dye with cresol red and tartrazine yellow. The amplification was performed in Arktik Thermal Cycler (Thermo Fisher Scientific, Vantaa, Finland). The following parameters were used for amplification: initial denaturation at 95°C for 3 min, 35 cycles of denaturation at 95°C for 50 s, annealing at 64°C for 50 s, and elongation at 72°C for 60 s, before a final elongation at 72°C for 3 min (Luk'yanchuk *et al*. 2018). PCR products were run at 200 V for 45 min on an electrophoresis gel (2.5% agarose (VWR International, Darmstadt, Germany), sodium borate buffer 10 mM NaOH and 36 mM H_3_BO_3_). To score for presence, gels were stained with GelRED (Biotium, Fremont, CA, USA), and size was compared using the standard GeneRuler 50 bp DNA Ladder (Thermo Fisher Scientific).

## RESULTS

### Resistance of *F. vesca* to *C. nymphaeae* shows considerable genetic variation

**Fig. 2:**
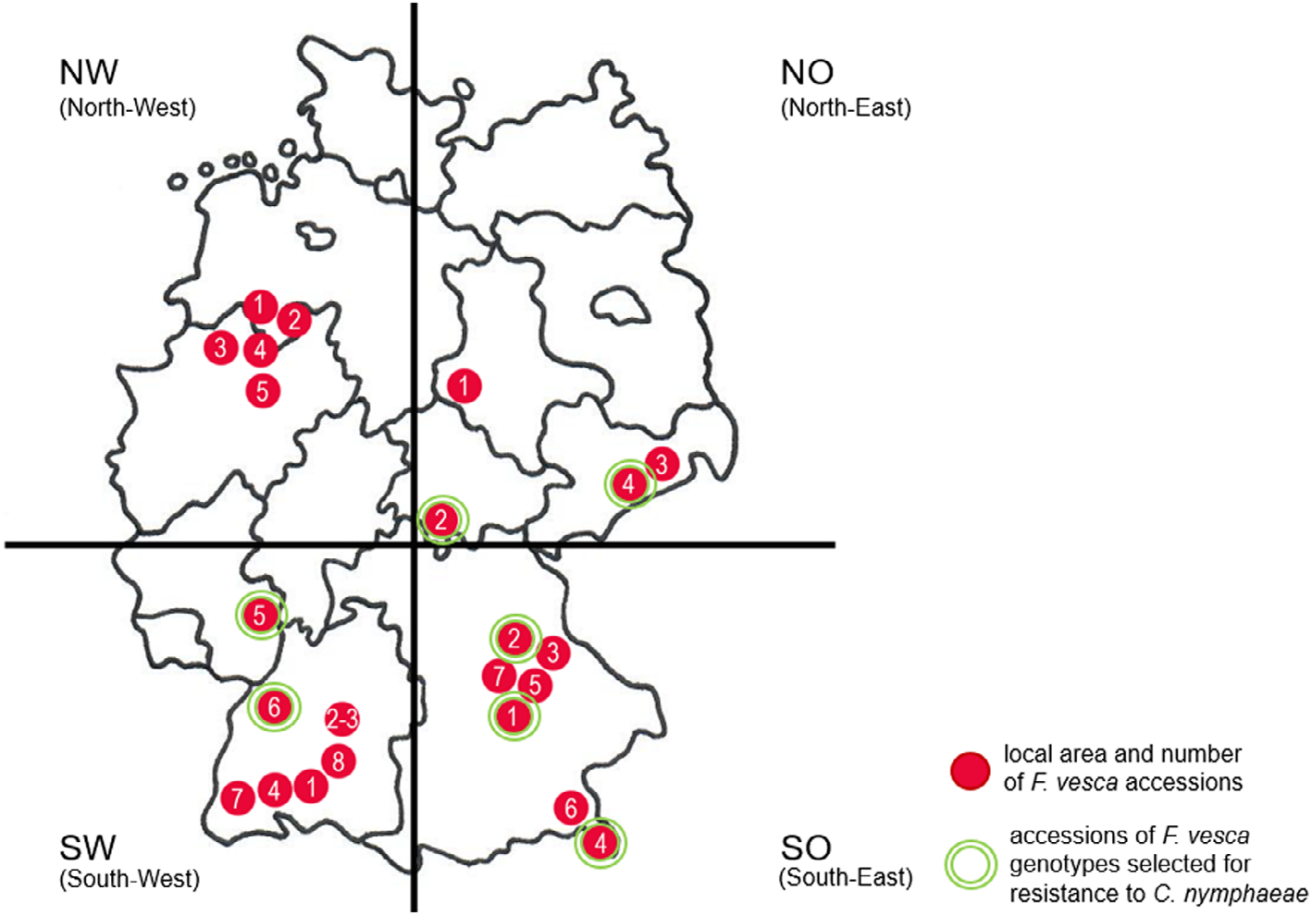
Geographical origin of *Fragaria vesca* accessions. North‐West (NW) accessions 01, 02, 03, 04, 05. North‐East (NO) accessions 01, 02, 03, 04. South‐West (SW) accessions 01, 02, 03, 04, 05, 06, 07, 08. South‐East (SO) accessions 01, 02, 03, 04, 05, 06, 07.

**Fig. 3:**
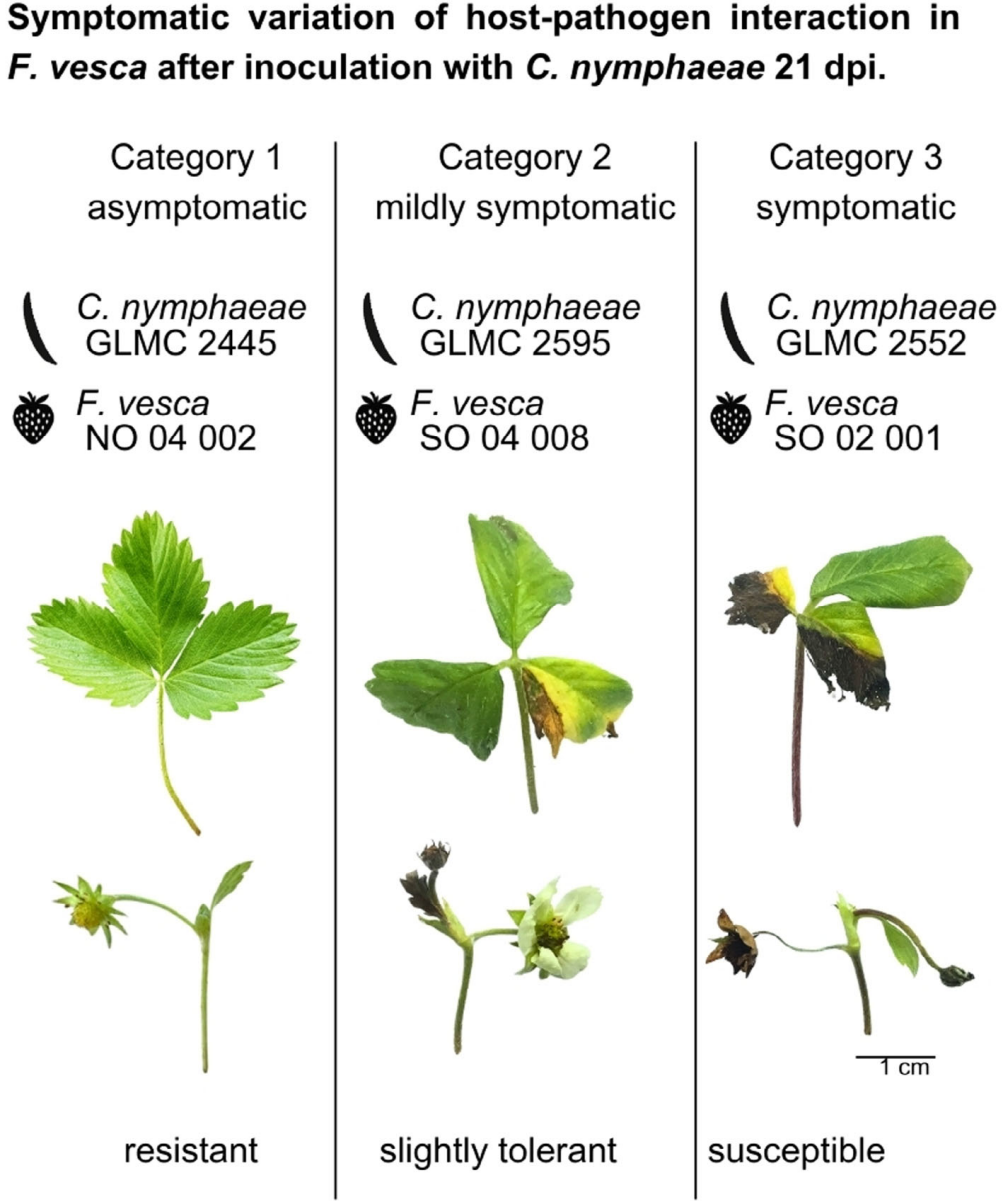
Symptoms in *Fragaria vesca* after inoculation with *Colletotrichum nymphaeae* 21 dpi.

During the vertical inoculation experiments at 21 days post‐inoculation (dpi) (Table 1), 30 of the 72 *F. vesca* genotypes showed no symptoms against *C. nymphaeae* GLMC 2445, 26 showed no symptoms against GLMC 2449, and nine showed no symptoms against GLMC 2455. Seven *F. vesca* genotypes from different geographic origins (Fig. 2), namely NO 02 001, NO 04 002, SO 01 022, SO 02 001, SO 04 008, SW 05 012 and SW 06 007, showed no symptoms against any of the three pathotypes (Table 1, category 1), and were considered as unspecific resistant. *F. vesca* genotypes tolerant to *C. nymphaeae* had necrotic lesions of category 2 (Table 1, Fig. 3). Susceptible *F. vesca* genotypes exhibited leaf lesions of category 3, which began at the edges and spread inward, forming oval to circular shapes that eventually coalesced. Dry, brittle, dark brown, necrotic tissue merged into a zone of lighter brown, sunken tissue, which merged into yellowed to healthy green tissue. Some necrotic tissue areas fell out (Table 1, Fig. 3). Heart leaves including stems, as well as runners and buds were also affected. In severe cases, complete necrotization of individual plant organs occurred until the highly susceptible plant died completely (Table 1, category 4). Susceptible genotypes were sorted out, except SO 03 007, which was chosen as contrasting partner for subsequent experiments. NO 03 005, NW 01 004 and SO 02 019 had strong symptoms against *C. nymphaeae* GLMC 2449 (category 3 to 4) but no symptoms against *C. nymphaeae* GLMC 2445 and GLMC 2455 (category 1), while NW 03 005, NW 05 037 and SO 06 018 showed strong symptoms against *C. nymphaeae* GLMC 2455 (category 3 to 4) but no symptoms against *C. nymphaeae* GLMC 2445 (category 1) (Table 1). Genotypes that showed resistance (category 1) or at least tolerance (category 2) to only one but not to other pathotypes of a species were considered to have specific resistance. Genotypes that showed susceptibility (category 3 to 4) to only one pathotype were considered to have specific susceptibility.

**Table 1:** *Fragaria vesca* symptomatic scoring against three *Colletotrichum nymphaeae* strains.

*Fragaria vesca genotype*	*Colletotrichum nymphaeae*				*Colletotrichum nymphaeae*				*Colletotrichum nymphaeae*		
	GLMC 2445	GLMC 2449	GLMC 2455	*Fragaria vesca genotype*	GLMC 2445	GLMC 2449	GLMC 2455	*Fragaria vesca genotype*	GLMC 2445	GLMC 2449	GLMC 2455
NO 01 005	2	2	2	NW 05 036	2	2	3	SW 01 017	2	2	3
NO 01 009	2	2	2	NW 05 037	1	2	3	SW 01 019	1	2	1
NO 01 010	2	2	2	NW 05 049	4	2	2	SW 01 020	2	2	2
NO 02 001	1	1	1	SO 01 022	1	1	1	SW 02 002	2	2	2
NO 02 002	2	2	2	SO 01 023	1	2	1	SW 02 003	2	2	2
NO 02 003	3	2	2	SO 01 025	1	2	1	SW 02 005	1	2	1
NO 03 003	1	2	2	SO 02 001	1	1	1	SW 03 011	1	2	2
NO 03 004	2	2	2	SO 02 002	2	2	2	SW 03 012	2	2	2
NO 03 005	1	3	1	SO 02 019	1	3	1	SW 03 009	2	3	2
NO 04 001	1	2	1	SO 03 005	2	2	2	SW 04 003	2	2	2
NO 04 002	1	1	1	SO 03 007	4	3	2	SW 04 004	1	2	1
NO 04 004	2	1	1	SO 03 008	1	2	1	SW 04 006	2	2	2
NW 01 001	1	2	2	SO 04 008	1	1	1	SW 05 010	2	2	2
NW 01 002	2	2	1	SO 04 012	1	2	1	SW 05 011	2	2	1
NW 01 004	1	4	1	SO 04 014	1	2	2	SW 05 012	1	1	1
NW 02 001	1	2	1	SO 05 018	2	2	2	SW 06 003	2	2	2
NW 02 002	2	2	2	SO 05 019	1	2	1	SW 06 004	2	2	3
NW 02 004	2	3	2	SO 05 020	1	2	1	SW 06 007	1	1	1
NW 03 003	3	2	2	SO 06 018	1	2	4	SW 07 002	2	2	2
NW 03 004	2	2	2	SO 06 020	2	1	1	SW 07 007	1	2	2
NW 03 005	1	2	3	SO 06 022	1	2	2	SW 07 011	1	2	1
NW 04 010	2	2	2	SO 07 001	1	2	2	SW 08 001	2	2	2
NW 04 011	2	2	2	SO 07 003	2	3	2	SW 08 003	2	2	2
NW 04 012	2	2	2	SO 07 004	2	2	2	SW 08 004	2	2	2

Categories: (1) no symptoms of infection, (2) single focus of infection, (3) strong infections on different parts of the plant, (4) died because of infection. Grey‐labelled genotypes showed resistance to all three pathotypes.

### Some host genotypes show multiple resistance to different *Colletotrichum* strains

Five of the seven vertical resistant *F. vesca* genotypes (NO 02 001, NO 04 002, SO 01 022, SO 04 008, SW 05 012) were found to be unspecific resistant or at least tolerant to further *C. nymphaeae* isolates (GLMC 2552, GLMC 2595, GLMC 2600) and repeatedly *C. nymphaeae* GLMC 2445 (Table 2). Regarding horizontal resistance, these exhibit diverse host–pathogen interactions to other *Colletotrichum* species, both within and between species (Table 2). The susceptible *F. vesca* genotype SO 03 007 repeatedly showed strong symptoms after inoculation with *C. nymphaeae* GLMC 2445 and minor symptoms with other *Colletotrichum* strains (Table 2). Control plants remained healthy throughout the observation period.

**Table 2:** *Fragaria vesca* symptomatic scoring against different *Colletotrichum* strains of various species.

*Fragaria vesca* genotype	*Colletotrichum nymphaeae*				*Colletotrichum godetiae*		*Colletotrichum lineola*	*Colletotrichum anthrisci*
	GLMC 2445	GLMC 2552	GLMC 2595	GLMC 2600	GLMC 2589	GLMC 2590	GLMC 2587	GLMC 2616
NO 02 001	1	1	1	2	1	2	1	1
NO 04 002	1	1	1	1	2	1	1	3
SO 01 022	1	2	1	1	1	2	2	2
SO 02 001	1	3	2	2	3	3	1	1
SO 04 008	1	1	2	1	2	1	1	2
SW 05 012	1	2	1	2	1	1	2	1
SW 06 007	1	2	3	2	1	1	2	2
SO 03 007	3	3	3	2	2	2	2	2

Categories: (1) no symptoms of infection, (2) single and small foci of infection, (3) strong infections on different parts of the plant, (4) died because of infection.

### Genotype‐specific expression of the symptomatic course of infection

In essence, *F. vesca* genotypes exhibited diverse responses to *Colletotrichum* strains, ranging from mild necrosis to complete tissue damage (Tables 1, 2, Fig. 3). Unique symptoms, such as a removable brown powder (NO 04 002), and typical reactions like black conidiomata or leaf curling were observed, highlighting the complexity of host–pathogen interactions.

*The susceptible variant SO 03 007* had some completely necrotized leaves and petioles after infection with *C. nymphaeae* GLMC 2552 and *C. godetiae* GLMC 2589. Necrosis on older leaves spread, as described for general symptoms, at *C. nymphaeae* GLMC 2595. Some necrotic tissue areas fell off, as with *C. nymphaeae* GLMC 2600 and *C. godetiae* GLMC 2590. Necrotic areas were partly covered with close, black, powdery spots. *C. anthrisci* GLMC 2616 caused a circular infection site that began to necrotize and was covered with irregular and patchy black spots. The infection with *C. lineola* GLMC 2587 showed only small necrotic tissue.

*NO 02 001* showed symptoms at *C. nymphaeae* GLMC 2600, occasionally severe necrosis on older leaves that spread, comparable to the above description of general symptoms. The necrotic tissue merged into a sharply defined, narrow zone of light brown, sunken tissue, which was followed by a wider, raised, yellow area which merged diffusely into healthy green tissue. This was accompanied by a longitudinal curling of the leaf blade. *C. godetiae* GLMC 2590 showed symptoms comparable to *C. nymphaeae* GLMC 2600.

*NO 04 002* showed symptoms for *C. nymphaeae* GLMC 2595 and GLMC 2600, with a rusty to dark brown, powdery coating formed on the vital green leaf tissue, which could be easily removed mechanically. Re‐isolation of the specific pathogen on culture medium from this brown powder was not successful. Sometimes there were dot‐like, black, raised structures. In interaction with *C. godetiae* GLMC 2589, symptoms developed on the leaf tip, comparable to NO 02 001. Symptoms of category 3 caused by *C. anthrisci* GLMC 2616 showed further formation of small, black, raised spots, identified as conidiomata. There was also tissue necrotization of heart leaves and young stolons.

*SO 01 022* showed symptoms at *C. nymphaeae* GLMC 2552 and *C. anthrisci* GLMC 2616 as described generally. Also *C. lineola* GLMC 2587 caused similar symptoms but with a significantly wider zone of light brown, sunken tissue between necroses and healthy green tissue. In interaction with *C. godetiae* GLMC 2590, only isolated small necroses of the leaf teeth were observed.

*SO 02 001* exhibited symptoms caused by *C. nymphaeae* GLMC 2552, GLMC 2595, and GLMC 2600, similar to those generally described. However, symptoms caused by GLMC 2600 featured a less pronounced transition zone. *C. godetiae* GLMC 2589 and GLMC 2590 developed stronger symptoms on leaves. *C. nymphaeae* GLMC 2552 and GLMC 2600 also caused necrotized buds and flower stalks, which also occurred with *C. godetiae* GLMC 2589 and GLMC 2590, as well as *C. anthrisci* GLMC 2616.

*SO 04 008* showed at *C. nymphaeae* GLMC 2595 leaf symptoms comparable to the general description, with necrotized buds and flower stalks. With *C. godetiae* GLMC 2589 there were necrotized young runners, and with *C. anthrisci* GLMC 2616 there was slight browning, with a diffuse transition covered with small black spots.

*SW 05 012* showed symptoms with *C. nymphaeae* GLMC 2552 and GLMC 2600 comparable to those generally found. With *C. lineola* GLMC 2587, a small site of infection was visible, with light brown necrotic tissue.

*SW 06 007* showed leaf symptoms comparable to the general symptoms at *C. nymphaeae* GLMC 2552, GLMC 2595, and GLMC 2600. *C. anthrisci* GLMC 2616 also showed similar symptoms in older leaves. *C. nymphaeae* GLMC 2552 additionally caused complete necrotized heart leaves.

With the exception of the brown, unidentified powder at NO 04 002 to *C. nymphaeae* GLMC 2595 and GLMC 2600, the pathogen strains were confirmed to be the cause of the symptoms through re‐isolation.

### Genotype *F. vesc*a NO 04 002 is highly resistant, while SO 03 007 is highly susceptible

In the comparative selection experiment, inoculation with *C. nymphaea* GLMC 2445 validated the categorization of the resistant genotype NO 04 002 from preliminary tests (Tables 1, 2) as category 1 in symptomatic scoring across all replicates (Table 3). The susceptible genotype SO 03 007 exhibited symptoms in all replicates, with eight plants dying (Table 3, categories 3 to 4). Symptom severity was significantly higher in SO 03 007 compared to the resistant NO 04 002 (Table 4). Control plants sprayed with sterile distilled water remained symptom‐free in both genotypes.

**Table 3:** Strength of infection.

*Fragaria vesca* genotype	category	rank	*Fragaria vesca* genotype	category	rank
NO 04 002	1	1	SO 03 007	4	13
NO 04 002	1	1	SO 03 007	4	13
NO 04 002	1	1	SO 03 007	4	13
NO 04 002	1	1	SO 03 007	3	11
NO 04 002	1	1	SO 03 007	3	11
NO 04 002	1	1	SO 03 007	4	13
NO 04 002	1	1	SO 03 007	4	13
NO 04 002	1	1	SO 03 007	4	13
NO 04 002	1	1	SO 03 007	4	13
NO 04 002	1	1	SO 03 007	4	13

Categories: (1) no symptoms of infection, (2) single and small foci of infection, (3) strong infections on different parts of the plant, (4) died because of infection.

**Table 4:** Infection area of contrasting *Fragaria vesca* genotypes through *Colletotrichum nymphaeae* GLMC 2445 12 hpi.

*Fragaria vesca* genotype	infection area mm^2^	*Fragaria vesca* genotype	infection area mm^2^
NO 04 002	0	SO 03 007	21.23
NO 04 002	0	SO 03 007	0.97
NO 04 002	0	SO 03 007	4
NO 04 002	0	SO 03 007	0
NO 04 002	0.3	SO 03 007	0
NO 04 002	0	SO 03 007	0
NO 04 002	0	SO 03 007	0
NO 04 002	0	SO 03 007	0
NO 04 002	‐	SO 03 007	0
NO 04 002	‐	SO 03 007	‐

For the resistant genotype NO 04 002, with 10 replicates, the Mann–Whitney *U* test yielded a *U*‐value of −45, based on a rank sum of 10. In contrast, the susceptible genotype SO 03 007, also with 10 replicates, had a rank sum of 126, resulting in a *U‐*statistic of 71. These results support the hypothesis that the two genotypes differ significantly in their symptom expression. The *U*‐statistic II values of 145 for the resistant NO 04 002, and 29 for the susceptible SO 03 007 do not influence this first statistical decision.

### Resistance in resistant *F. vesca* genotype NO 04 002 correlates with a much weaker NBT signal than in the susceptible SO 03 007 genotype

**Fig. 4:**
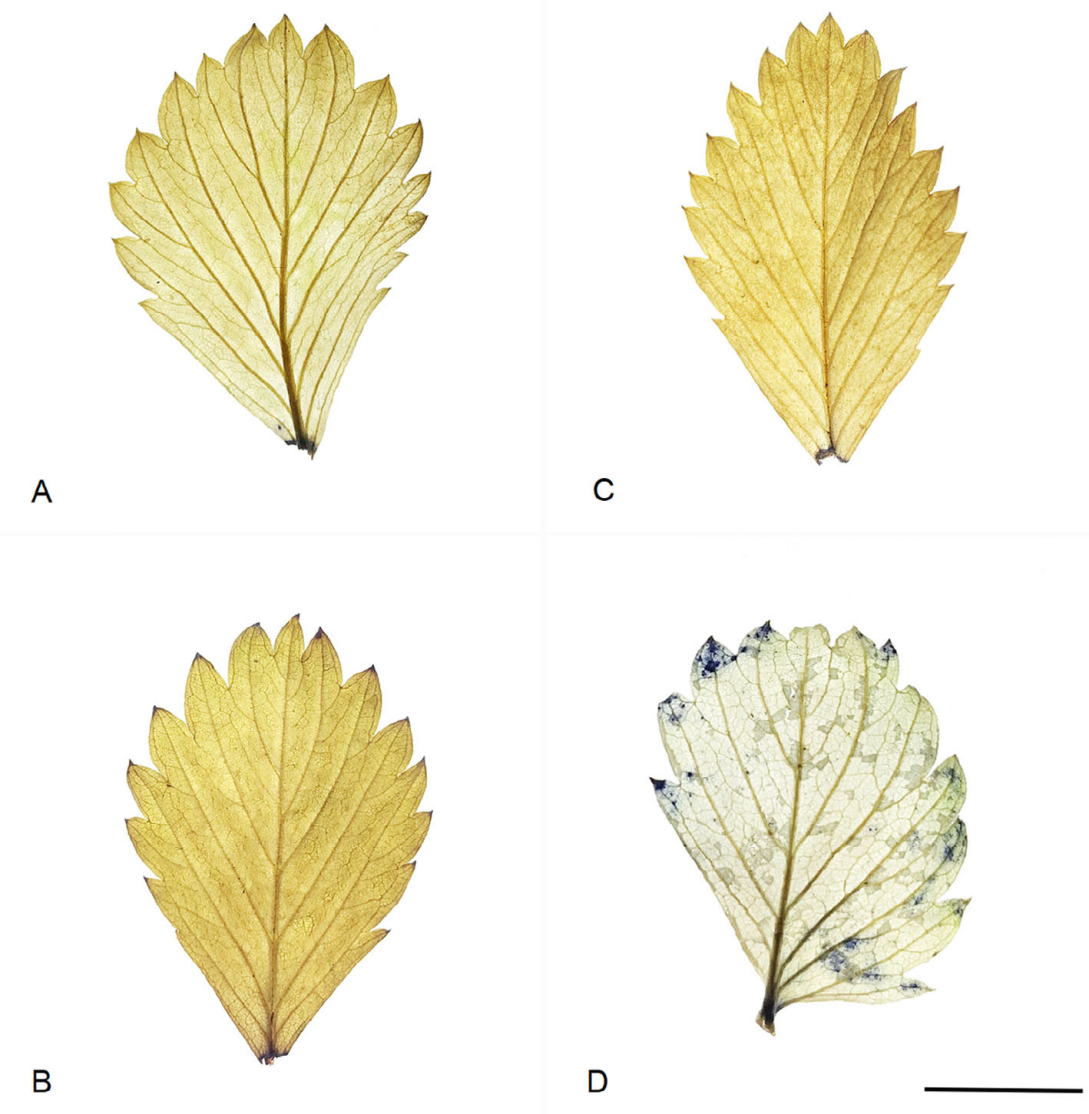
Superoxide production in *Fragaria vesca* through *Colletotrichum nymphaeae* GLMC 2445 12 hpi in extreme. (A and B) NO 04 002. (C and D) SO 03 007. (A and C) Control. (B and D) Inoculated. (A–D) After NBT treatment and ethanol discoloration. Scale bar: D = 1 cm. Scale bar of D applies to A–D.

The NBT test provided evidence of different host–pathogen interactions between the resistant *F. vesca* genotype NO 04 002 and the susceptible SO 03 007 against *C. nymphaeae* GLMC 2445. The resistant *F. vesca* genotype NO 04 002 showed no blue discoloration in seven of eight repetitions. In one leaf sample, the tissue showed a light, diffuse bluish discoloration, with a total area of 0.3 mm^2^ 12 hpi (hours post‐inoculation) (Fig. 4B, Table 4). Immediately, there was no discoloration of the NBT solution visible upon contact with the sampled tissue. After contact with infected plant material of the susceptible genotype SO 03 007, the test solution reacted with a purple coloration. After NBT treatment, the susceptible genotype SO 03 007 showed (in one sample) strong development of blue coloration around the infection spot at 12 hpi covering a 21 mm^2^ infected leaf area (Fig. 4D), and 1 mm^2^ and 4 mm^2^ in two further samples (Table 4). The smaller infection area of the resistant genotype NO 04 002 (Table 4) resulted in a lower mean value (0.04) compared to the susceptible SO 03 007 (2.91) (Table S4). Six samples of SO 03 007 showed no spots. Two samples of the resistant NO 04 002 and one sample of susceptible SO 03 007 genotype were excluded from the analysis due to poor material quality after the ethanol treatment. All the control plants that were sprayed with sterile, distilled water showed no discoloration. These visual difference in discoloured tissue of the resistant *F. vesca* genotype NO 04 002 and the susceptible genotype SO 03 007 (Fig. 4) could not be supported statistically (Table S3). Differences were not statistically significant, since *P* (0.13) > *α* (0.05) and critical *t‐*value (1.86) > *t*‐statistic (−1.23). Hence, H_0_, which states that there are no differences, must be accepted.

### Resistance in the resistant genotype NO 04 002 correlates with much weaker SA accumulation than in the susceptible SO 03 007

The mixed sample of the resistant genotype NO 04 002 showed an increase in total SA (279%) and free SA (460%) 12 hpi with strain *C. nymphaeae* GLMC 2445 compared to the control. In contrast, the mixed sample of the susceptible genotype SO 03 007 showed a more extreme increase in total SA (3531%) and free SA (517%) after the same treatment (Table S4). The total SA content decreased in SO 03 007 and increased slightly in NO 04 002 over the following 2 days. The samples were near the level of detection (LOD) due to the small amount of material available.

### Resistance gene *RCA2* seems absent from the sampled *F. vesca* genotypes

Using PCR, we tested the resistant *F. vesca* genotypes NO 02 001, NO 04 002, SO 01 022, SO 02 001, SO 04 008, SO 06 020, SW 05 012, SW 06 007, and the susceptible genotype SO 03 007, as well as *F. × ananassa* cultivars ‘Elsanta’ and ‘Asia’ negative for the presence of the resistance gene *RCA2*. The positive control *F. × ananassa* ‘Capitola’ was confirmed positive for the gene (Fig. S1).

## DISCUSSION

This study highlights the high genetic diversity in *F. vesca* against economically important *Colletotrichum* pathotypes in Germany. We observed multiple host–pathogen interactions through successive inoculation experiments, and one *F. vesca* genotype (NO 04 002) with broadresistance against *C. nymphaeae* (Figs. 1 and 3, Tables 1 and 2). Due to its nonspecific resistance, it is suspected that the genotype NO 04 002 relies on basal immunity. This suggests that host and pathogen did not undergo co‐evolution. Contrary to general expectations of basal immune responses (PTI), we identified more active accumulation of superoxide at the infection site and a strongly increased SA concentration in the susceptible genotype SO 03 007 (Fig. 4).

### *Colletotrichum*‐mediated cell death is triggered by reactive oxygen species (ROS)

In preparatory tests, no superoxide production was detected after 4 h of incubation (data not shown), an incubation period recommended for cultivated strawberry (Grellet‐Bournonville & Díaz‐Ricci 2011). For *F. vesca*, we were able to achieve visible success after an incubation period of 12 h. Since a rapid immune response of ETI is activated after only a few minutes and is completed after a few hours (Grellet‐Bournonville & Díaz‐Ricci 2011), it must be suspected that after 12 h only late defence of basal immune responses could be detected, which could explain the slight diffuse blue discoloration in the resistant genotype NO 04 002 (Fig. 4). At this point, defence against the fungus was probably completed, without symptomatic consequences. Ma *et al*. (2022) suggested that pathogen‐induced ROS must reach a specific threshold to cause cell death. The increased superoxide production of the susceptible genotype SO 03 007 might be a consequence of programmed cell death, triggered by immune responses of illegitimate HR (Ma *et al*. 2022) (Fig. 4). Sometimes pathogens induce HR in the biotrophic phase, causing the host cell to kill itself. As a result, the pathogen colonizes and proliferates in the host upon transition to the necrotrophic phase. The time to immune‐mediated symptoms, even cell death, can vary greatly and may take several days (Teper *et al*. 2020; Ma *et al*. 2022) (Fig. 3, Table 3). An illegitimate HR is currently known from a bacterium that causes Huangbinglong (HLB) disease, one of the most devastating citrus diseases worldwide (Wang 2019; Ma *et al*. 2022). We would therefore have observed a chronic, systemic immune response in *F. vesca*. However, to draw clear conclusions, it is essential to determine the life phase of the pathogen, biotrophic or necrotrophic, during the ROS measurements. When searching for resistant genotypes, ROS‐suppressing factors should be selected that reduce cell death and thereby alleviate symptoms (Ma *et al*. 2022). For our resistant genotype NO 04 002, we suspect that it prevents the fungus from emitting its manipulation signal through strong basal immunity.

### Salicylic acid increases as a result of infection

When mediating plant defence against biotic or abiotic stress, SA values increase, usually attributed to an ETI (Grellet‐Bournonville *et al*. 2012). In many plants, activators of SA‐dependent defence are known to be important downstream components of the SA signalling pathway and were detected in garden strawberry as a pathogen‐specific reaction to a *Colletotrichum* infection (Grellet‐Bournonville *et al*. 2012). In cultivated strawberry, kinases, phosphatases and genes (*FaEDS1* and *FaPAD4*; *FaWRKY70‐1* and *FaWRKY70‐2*; *FaMeSA1*; *FaPBS1*; *FaGRX1*; *FaPR1‐2*, *FaPR2‐1* and *FaPR2‐2*) are involved in mediating resistance (Amil‐Ruiz *et al*. 2016). It is even suspected that the gene *M9D5EST*, which encodes a methylsalicylate esterase, is involved in the defence process of strawberries against *Colletotrichum* (Amil‐Ruiz *et al*. 2016). In Zhang *et al*. (2016), tolerant strawberry varieties showed higher basal SA content, which inhibited conidial germination. These authors compared their own results of a *C. gloeosporioides* infection on susceptible cultivated strawberry with external studies on wild *F. vesca* genotypes, whereby the resistance gene *FaNBS30* increased slowly in the cultivated variety, whereas in *F. vesca* there was no increase of *FvNBS30* (Zhang *et al*. 2016). Due to numerous irregularities in the NB‐LRR homologues of octo‐ and diploid *Fragaria* species, they concluded that the expression patterns of the NB‐LRR genes are complex (Zhang *et al*. 2016).

Our investigations determined increased SA content after infection with *C. nymphaeae* GLMC 2445 in resistant *F. vesca* NO 04 002, but this was much more extreme in the susceptible genotype SO 03 007 (Table S4). Perhaps another sign of an illegitimate HR. However, it should be noted that the observed increase in SA content of both genotypes fluctuated over time, and the measurement results obtained were very close to the detection limits (Table S4).

### Limited co‐evolutionary development

In Germany, due to spatial and/or temporal separation of invasive *Colletotrichum* isolates in the field (Rose & Damm 2024) and the *F. vesca* populations in the landscape, we suggest little to no co‐evolutionary development of monogenetic resistances (ETI). However, it is important to note that the collection described by Rose & Damm (2024) focused exclusively on *F. × ananassa* cultivated in fields. To date, no systematic sampling of *Colletotrichum* species, particularly in *F. vesca* populations in natural habitats, has been conducted in Germany. This study highlights the potential risk of *Colletotrichum* species being transmitted from infected field plants into natural ecosystems, where they could initiate a co‐evolutionary process leading to Effector‐Triggered Immunity (ETI) in native *Fragaria* species, like *F. vesca*.

*Colletotrichum nymphaeae*, showed largely genetic stability (Rose & Damm 2024), which supports co‐evolutionary development and the formation of monogenic resistance in garden strawberry and perhaps with North American *F. vesca* genotypes. We consider co‐evolutionary development with German *F. vesca* unlikely due to long spatial separation, although *C. nymphaeae* is the most frequent species in Germany and has already been found once on *F. vesca var. semperflorens*, in Hesse, Germany (Rose & Damm 2024). Even if there is no more precise information about where the isolate was found, we assume that this cultivated form of a wild strawberry was also in a cultivated environment and was infected *ex situ*. However, in Brandenburg, Germany, *Colletotrichum* was currently found in *Nymphaea* from a natural area identified by morphological characteristics probably as *C. nymphaeae* (Dämmrich *et al*. 2023).

The presence of *C. godetiae* in strawberry fields is characterized by temporal and spatial separation from *F. vesca*, ruling out the possibility of co‐evolutionary development. In contrast, *C. lineola*, which is known to infect various plants, including strawberries, across the Northern Hemisphere (Damm *et al*. 2009; Tsvetkova & Kuznetsova 2022) could potentially be transferred between hosts. However, as *C. lineola* was last reported in German strawberry fields over 20 years ago, its spread from cultivated fields into natural ecosystems is considered unlikely.

*Colletotrichum anthrisci* was found on *F. vesca* in a forest in Saxony, Germany, causing leaf spots (Rose & Damm 2024). The genotype NO 04 002, resistant to six *C. nymphaeae* strains, reacted to *C. anthrisci* GLMC 2616 with symptoms of severity of up to 3 (Table 2). Since this host–pathogen interaction was found only recently and only determined once *in situ* (Rose & Damm 2024), no definitive statements are made as to earlier co‐evolutionary development. However, the transmission of *F. vesca* to cultivated strawberry *F*. × *ananassa* ‘Asia’ was successful in the laboratory (Rose & Damm 2024). A future co‐evolutionary development and the development of specific resistance might be more probable.

### Consideration of the diverse immune strategies in breeding programmes

In *F. × ananassa*, resistance to *Colletotrichum* is based on ETI. Monogenetic resistance is only effective if the dominant resistance gene and dominant avirulence gene are paired (Denoyes‐Rothan *et al*. 2005). Through breeding, single genes are easy to place into the genome of a crossing partner. Because of the high pathogen diversity and strong interaction with strawberries, we expect monogenic resistance to be rapidly overcome. Among cultivated strawberries, susceptibility is caused through the lack of a dominant resistance gene due to recessive homozygosity (Denoyes‐Rothan *et al*. 2005). To support marker‐assisted selection of this trait, SCAR markers have been developed on the basis of an AFLP‐based analysis of a cross between the resistant ‘Capitola’ and the susceptible ‘Pajaro’ (Lerceteau‐Köhler *et al*. 2005). Validation of these markers in a core set of strawberry varieties showed that around ¾ of the anthracnose‐resistant genotypes correlated with the presence of the *RCA2* locus, as reported by these SCAR markers. However, in a quarter of genotypes, the observed resistance was independent of *RCA2*, which can be explained by di‐ to tetrasomic effects (Denoyes‐Rothan *et al*. 2005; Lerceteau‐Köhler *et al*. 2005).

There are also reports of low heterozygosity in *F. vesca* and descriptions of resistance against various diseases through major genes (Denoyes‐Rothan *et al*. 2005; Eikemo *et al*. 2010; Zhou *et al*. 2023). Resistance to *C. acutatum* was even suspected to be homozygous (Denoyes‐Rothan *et al*. 2005). Ultimately, it can be said that the gene *RCA2* was not involved in resistance of our selected *F. vesca* genotypes (Fig. S1). The results of our studies suggested the presence of additional, possibly basal immune responses in *F. vesca*. This highlights the value of the selected genotypes, particularly NO 04 002, as promising candidates for resistance breeding. Because of their effectiveness against various pathotypes, we recommend basal immunity of CWR for sustainable breeding (Tomooka *et al*. 2000; Tullu *et al*. 2006; Karki *et al*. 2021; Cason *et al*. 2022), which requires consideration of different modes of inheritance when designing breeding programmes. Denoyes‐Rothan *et al*. (2005) derive from the quantitative resistance a permanent defence capacity of strawberry plants and even of fruits. To cross the immune properties of the diploid *F. vesca* into the octoploid *F*. × *ananassa*, different ploidy levels must be overcome to obtain fertile crossing partners. For that, mitosis inhibitors cause a multiplication of the chromosome set and thus polyploidy.

### Minor role of geographic origin

Resistance to *C. nymphaeae* in *F. vesca* genotypes was largely independent of their geographic origin within Germany, although not all areas were sampled with the same intensity (Fig. 2). In agreement, large intraspecific variations in resistance were found for *F. vesca* against the pathogen *P. cactorum*, also independent of the geographic host origin (Eikemo *et al*. 2010). In octoploid *F. virginiana* and *F. chiloensis*, no subspecies or geomorph was more resistant to *C. fragariae*, *C. gloeosporioides* and *C. acutatum* than the others, but again between individual genotypes (Lewers *et al*. 2007).

## CONCLUSIONS

In Europe, current resistance strategies in strawberry against anthracnose primarily target monogenetic resistances to *C. acutatum*. However, this focus may be misleading due to historically unreliable pathogen identification methods, which often misclassified *Colletotrichum* species. To address this gap, we concentrated on *C. nymphaeae* in our studies, as molecular data now identify it as a major pathogen in German strawberry fields. We further distanced ourselves from the usual breeding focus on monogenic resistance in cultivated strawberries and looked for PTI in CWR *F. vesca* to counteract gene erosion. Our analysis of 72 *F. vesca* genotypes revealed high diversity of host–pathogen interactions. This approach shows promise for resistance against multiple pathogens, a shift towards basal initiated immunity. Most surprising was the result that the *F. vesca* genotype SO 03 007, susceptible to *C. nymphaeae*, reacted with strong histochemical defence, which is normally typical for a successful pathogen defence of resistant genotypes. It is concluded that anthracnose is an immune‐mediated disease resulting from ROS‐induced cell death. In *F. vesca*, an illegitimate HR can be assumed as an immune response. We also found that resistance is more heavily influenced by the plant than its geographic origin, challenging the traditional focus on location‐specific breeding. We support the use of CWR in novel breeding programmes to develop robust strawberry varieties capable of withstanding diverse pathogenic pressures, ultimately enhancing sustainability and productivity in strawberry cultivation.

## AUTHOR CONTRIBUTIONS

CR conducted the experiments together with IV. All authors contributed equally to writing the paper.

## Supporting information


**Table S1.** Accessions of *Fragaria* [*F*.] *vesca*, with collection data and identification numbers.
**Table S2.** Primer sequences of AccuStart II PCR SuperMix (Quantabio, USA).
**Table S3.** Two‐sample *t*‐test assuming different variances in superoxide production of contrasting *F. vesca* SO 03 007 and NO 04 002 after inoculation with *C. nymphaeae* 2445 in NBT test.
**Table S4.** SA levels after 1, 2, 3 dpi (days post inoculation) with *C. nymphaeae* of the *F. vesca* genotypes NO 04 002 (resistant) and SO 03 007 (susceptible).
**Fig. S1.** Gel image for detection of the *RCA2* and EMF genes in *F. × ananassa* and *F. vesca* genotypes.
