# Supplementary material for: The crop wild relative Fragaria vesca as source of resistance against strawberry anthracnose
Source: Plant Biol (Stuttg). 2025 Aug 7;27(7):1353–65. doi: 10.1111/plb.70087 (PMC12631507; doi:10.1111/plb.70087)
Supplement: Supplementary file 1 — Table S1. Accessions of Fragaria [F.] vesca, with collection data and identification numbers. Table S2. Primer sequences of AccuStart II PCR SuperMix (Quantabio, USA). Table S3. Two‐sample t‐test assuming different variances in superoxide production of contrasting F. vesca SO 03 007 and NO 04 002 after inoculation with C. nymphaeae 2445 in NBT test. Table S4. SA levels after 1, 2, 3 dpi (days post inoculation) with C. nymphaeae of the F. vesca genotypes NO 04 002 (resistant) and SO 03 007 (susceptible). Fig. S1. Gel image for detection of the RCA2 and EMF genes in F. × ananassa and F. vesca genotypes. [file PLB-27-1353-s001.docx]

| **Sup. Tab. 1.** Accessions of *Fragaria* [*F*.] *vesca*, with collection data and identification numbers. | | | | |
| --- | --- | --- | --- | --- |
| **Locality** | **Origin** | **Stock code - Collection date** | **Collectors code** | **Breeders code** |
| Baden-Württemberg, | WEL | SW-03-0400 - 2012 | 3 400 2012 | SW 02 002 |
| Reutlingen, |  |  |  | SW 02 003 |
| Mittlere Kuppenalb |  |  |  | SW 02 005 |
| Baden-Württemberg, | WEL | SW-05-0431 - 2013 | 5 431 2013 | SW 03 009 |
| Reutlingen, |  |  |  | SW 03 011 |
| Mittlere Kuppenalb |  |  |  | SW 03 012 |
| Baden-Württemberg, | WEL | SW-05-0398 - 2013 | 5 398 2013 | SW 08 001 |
| Zollernalbkreis, |  |  |  | SW 08 003 |
| Hohe Schwabenalb |  |  |  | SW 08 004 |
| Baden-Württemberg, | WEL | SW-03-0509 - 2013 | 3 509 2013 | SW 04 003 |
| Schwarzw.-Baar-Kreis, |  |  |  | SW 04 004 |
| Südöstlicher |  |  |  | SW 04 006 |
| Schwarzwald |  |  |  |  |
| Baden-Württemberg, | WEL | SW-03-0502 - 2013 | 3 502 2013 | SW 05 010 |
| Rhein-Neckar-Kreis, |  |  |  | SW 05 011 |
| Bergstrasse |  |  |  | SW 05 012 |
| Baden-Württemberg, | WEL | SW-03-0217 - 2011 | 3 217 2011 | SW 06 003 |
| Rastatt, |  |  |  | SW 06 004 |
| Nördlicher |  |  |  | SW 06 007 |
| Talschwarzwald |  |  |  |  |
| Baden-Württemberg, | WEL | SW-05-0447 - 2013 | 5 447 2013 | SW 01 017 |
| Sigmaringen, |  |  |  | SW 01 019 |
| Baaralb und |  |  |  | SW 01 020 |
| Oberes Donautal |  |  |  |  |
| Baden-Württemberg, | WEL | SW-03-0508 - 2013 | 3 508 2013 | SW 07 002 |
| Freudenstadt, |  |  |  | SW 07 007 |
| Mittlerer Schwarzwald |  |  |  | SW 07 011 |
| Bavaria, | WEL | SO-01-0649 - 2012 | 01 0649 2012 01 | SO 07 001 |
| Neumarkt i. d. OPf., |  |  |  | SO 07 003 |
| Südliche Frankenalb |  |  |  | SO 07 004 |
| Bavaria, | WEL | SO-01-0034 - 2010 | 01 0034 2010 01 | SO 02 001 |
| Neumarkt i. d. OPf., |  |  |  | SO 02 002 |
| Mittlere Frankenalb |  |  |  | SO 02 019 |
| Bavaria, | WEL | SO-01-1031 - 2013 | 01 1031 2013 01 | SO 06 018 |
| Berchtesgad. Land, |  |  |  | SO 06 020 |
| Berchtesgad. Alpen |  |  |  | SO 06 022 |
| Bavaria, | WEL | SO-01-0253 - 2011 | 01 0253 2011 01 | SO 04 008 |
| Berchtesgad. Land, |  |  |  | SO 04 012 |
| Berchtesgad. Alpen |  |  |  | SO 04 014 |
| Bavaria, | WEL | SO-01-0208 - 2011 | 01 0208 2011 01 | SO 03 005 |
| Schwandorf, |  |  |  | SO 03 007 |
| Falkensteiner Vorwald |  |  |  | SO 03 008 |
| Bavaria, | WEL | SO-01-0208 - 2011 | 01 0208 2011 01 | SO 03 005 |
| Schwandorf, |  |  |  | SO 03 007 |
| Falkensteiner Vorwald |  |  |  | SO 03 008 |
| Bavaria, | WEL | SO-01-0033 - 2010 | 01 0033 2010 01 | SO 01 022 |
| Kelheim, |  |  |  | SO 01 023 |
| Südliche Frankenalb |  |  |  | SO 01 025 |
| Bavaria, | WEL | SO-01-0653 - 2012 | 01 0653 2012 01 | SO 05 018 |
| Regensburg, |  |  |  | SO 05 019 |
| Südliche Frankenalb |  |  |  | SO 05 020 |
| North Rhine-Westphalia, | WEL | NW-00-0084 - 2016 | 16 00 0084 | NW 03 003 |
| Steinfurt, |  |  |  | NW 03 004 |
| Osnabrücker Hügelland |  |  |  | NW 03 005 |
| Lower Saxony, | WEL | NW-03-0010 - 2009 | 03 0010 2009 | NW 05 036 |
| Osnabrück, |  |  |  | NW 05 037 |
| Ostmünsterland |  |  |  | NW 05 049 |
| Lower Saxony, | WEL | NW-00-0474 - 2016 | 16 00 0474 | NW 01 001 |
| Osnabrück, |  |  |  | NW 01 002 |
| Osnabrücker Hügelland |  |  |  | NW 01 004 |
| Lower Saxony, | WEL | NW-03-0212 - 2012 | 03 0212 2012 | NW 02 001 |
| Osnabrück, |  |  |  | NW 02 002 |
| Westl.Wiehengebirge |  |  |  | NW 02 004 |
| (Wittlager Eggen) |  |  |  |  |
| Lower Saxony, | WEL | NW-00-0419 - 2016 | 16 00 0419 | NW 04 010 |
| Osnabrück, |  |  |  | NW 04 011 |
| Tecklenburger Osning |  |  |  | NW 04 012 |
| Saxony-Anhalt, | WEL | NO-01-0425 - 2011 | DE 0 8 1390911 | NO 01 005 |
| Unterharz |  | NO-01-0428 - 2011 |  | NO 01 009 |
|  |  |  |  | NO 01 010 |
| Thuringia, | C. Rose | TH 01 - 2019 | 1 2019 | NO 02 001 |
| Kloster Veßra, |  |  |  | NO 02 002 |
| Schleusemündung |  |  |  | NO 02 003 |
| Saxony, | C. Rose | KG 01 - 2019 | 3 2019 | NO 03 003 |
| Dresden, |  |  |  | NO 03 004 |
| Pillnitz |  |  |  | NO 03 005 |
| Saxony, | C. Rose | WH 01 - 2019 | 00 2019 | NO 04 001 |
| Sächs. Schweiz- |  |  |  | NO 04 002 |
| Osterzgeb. |  |  |  | NO 04 004 |
| Bannewitz |  |  |  |  |
| Genbank für Wildpflanzen für Ernährung und Landwirtschaft (WEL), Botanischer Garten Osnabrück, Germany; C. Rose, Hochschule Geisenheim University, Germany; Breeders Code: NW, North-West; NO, North-East; SW, South-West; SO, South-East as abbreviation of the points of compass for subdivide the federal territory of Germany added by number of local area and genotype. | | | | |

**Sup. Tab. 2.** Primer sequences of AccuStart II PCR SuperMix (Quantabio, USA)

Sequence from 5’ to 3’

STS-Rca2_240-F gccacgtcactagtcaaattcaa

STS-Rca2_240-R tcttggacagtggtctcagc

EMFv020-F caggcgccaacggcgtgctcttgt

EMFv020-R cagcgccgccagctcatccctagg

**Sub. Tab. 3.** Two-sample T-test assuming different variances in superoxide production of contrasting *F. vesca* SO 03 007 and NO 04 002 after inoculation with *C. nymphaeae* 2445 in NBT test.

| **Statistical parameters** | **Infection area mm^2^** | |
| --- | --- | --- |
|  | **NO 04 002** | **SO 03 007** |
| Mean value | 0,0375 | 2,911111111 |
| Variance | 0,01125 | 48,92283611 |
| Observations | 8 | 9 |
| Hypothetical difference in means | 0 |  |
| Degrees of freedom (df) | 8 |  |
| t-statistic | -1,232359076 |  |
| P(T<=t) one-sided | 0,126401297 |  |
| Critical t-value for one-tailed t-test | 1,859548038 |  |

**Sub. Tab. 4.** SA levels after 1, 2, 3 dpi (days post inoculation) with *C. nymphaeae* of the *F. vesca* genotypes NO 04 002 (resistant) and SO 03 007 (susceptible)

| **dpi** | **treatment** | **Genotype** | **total weight (mg)** | **total SA** | **free SA** | **SA-Gluc** |
| --- | --- | --- | --- | --- | --- | --- |
| 1 | H_2_O | NO 04 002 | 98,9 | 0,33437512 | 0,6662554 | 0* |
| 1 | H_2_O + pathogen | NO 04 002 | 85,3 | 0,91559818 | 3,07547824 | 0* |
| 1 | H_2_O | SO 03 007 | 78,1 | 0,7835974 | 2,40423453 | 0* |
| 1 | H_2_O + pathogen | SO 03 007 | 73,9 | 27,5410091 | 12,4040319 | 15,1369771 |
| 2 | H_2_O | NO 04 002 | 102,7 | 6,27928378 | 0,11420111 | 6,16508267 |
| 2 | H_2_O + pathogen | NO 04 002 | 94,5 | 0,59182874 | 1,98043703 | 0* |
| 2 | H_2_O | SO 03 007 | 94,5 | 10,8101332 | 3,65523275 | 7,15490042 |
| 2 | H_2_O + pathogen | SO 03 007 | 90,3 | 6,25078886 | 5,66342001 | 0,58736885 |
| 3 | H_2_O | NO 04 002 | 82,5 | 16,4474623 | 1,15801768 | 15,2894446 |
| 3 | H_2_O + pathogen | NO 04 002 | 86,2 | 5,44936331 | 0,82865875 | 4,62070455 |
| 3 | H_2_O | SO 03 007 | 88,3 | 3,62659049 | 0,94014497 | 2,68644551 |
| 3 | H_2_O + pathogen | SO 03 007 | 84,4 | 1,28769707 | 10,3418275 | 0* |

Total weight = mixed sample of 10 replicates per genotype; SA (salicylic acid); total SA = acid hydrolysis; SA-Gluc = total SA – free SA (*negative values set as '0').


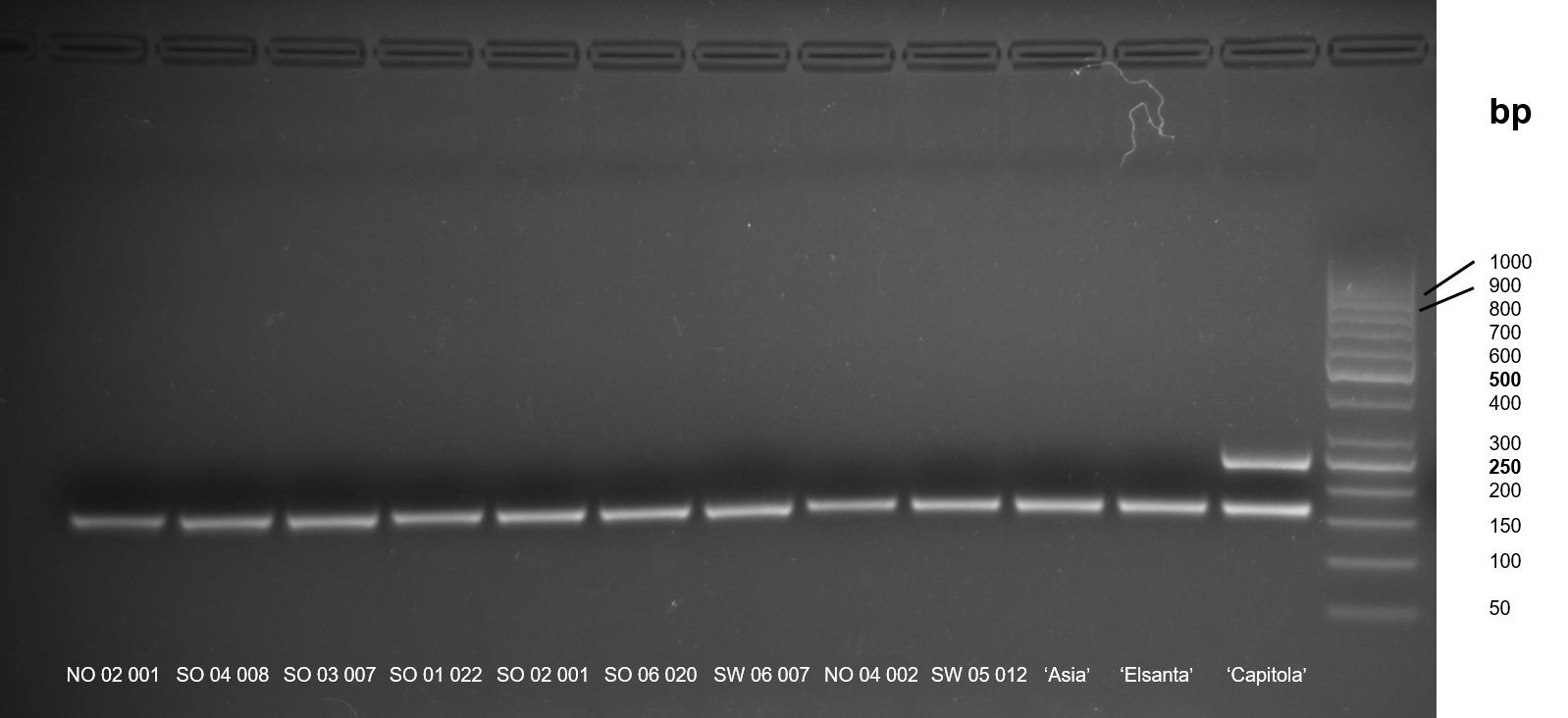


**Sup. Fig. 1.** Gel image for detection of the *RCA2* and EMF genes in *F. ×* *ananassa* and *F. vesca* genotypes.
